# Predictors for Sexual Intercourse Experience among Runaway Female At-Risk Adolescents

**DOI:** 10.3390/ijerph17113913

**Published:** 2020-06-01

**Authors:** So-Hyun Moon, Hyung-Ran Kim, Miok Kim

**Affiliations:** 1 Department of Nursing, College of Medicine, Chosun University, Gwangju 61452, Korea; shmoon@chosun.ac.kr; 2 Department of Pediatrics, Kwangju Christian Hospital, Gwangju 61661, Korea; brood1982@naver.com; 3 Department of Nursing, College of Nursing, Dankook University, Cheonan 31116, Korea

**Keywords:** adolescents, behavior, environment, logistic models

## Abstract

This study attempted to provide basic data for creating a program to help promote safe sexual behavior among runaway female at-risk adolescents by identifying factors related to the sexual experiences. This study conducted a logistic regression analysis using data regarding 182 female at-risk adolescents, which were sourced from the 2016 survey of Korean adolescents’ contact with media usage and harmful environment. This study showed that adolescents’ age, smoking, and harmful environments are associated with the occurrence of sexual activity among at-risk female adolescents. One significant outcome of this study was the identification of harmful environmental factors and their impact on sexual behavior. Since smoking and sex-related problems among adolescents can act as risk factors for adult sexual health in the future, schools should institute direct and indirect channels for assessing sex-related problems among runaway female at-risk adolescents and establishing proactive and preventive measures for promoting their sexual health. In addition, a social cooperation system should be established in order to assess, and mediate within, the environments around schools in order to minimize adolescents’ exposure to harmful environments.

## 1. Introduction

An “at-risk adolescent” can be defined as a young person aged from 10 to 18 years who experiences difficulties in studying or practicing social adaptation due to family problems, economic problems, or psychological disorders [1], that is, “those who are not be able to get a job or achieve a full life as adults due to their inadequate adaptation to school life, and as a result, adolescents who are unlikely to contribute enough to society” [2]. In 2016, at-risk adolescents were estimated to form 17.7% of South Korean adolescent population (about 780,000), and this trend has continued; the percentage of such adolescents increased from 17.6% of high risk groups and 27.3% of potential risk groups, in 2010, to 19.3% and 29.8%, respectively, of such groups in 2015 [1]. This has led to diversified efforts aimed at establishing at-risk adolescent management systems.

At-risk adolescents often become runaways who have run away from their family or institutions due to personal and/or family problems or difficulties in adapting to school. Their runaway rate has been reported to be 69.8%, which is higher than that of ordinary adolescents (11%) [3]. Runaway adolescents are more likely to experience mental health problems such as depression, self-harming behaviors, and suicidal attempts [4]; they are also more susceptible to problems such as academic interruption, theft aimed at making a livelihood [5], and misconducts related to physical assaults [6]. In addition, runaway adolescents are more vulnerable to negative sexual health outcomes because they tend to be more sexually active than non-at-risk adolescents, and thus are more likely to be exposed to risky behaviors such as having multiple sexual partners, participating in unprotected sex, and getting lured into the sex trade; they also face a higher risk of contracting HIV and STIs [7]. Runaway adolescents also commonly face drug and alcohol problems [8], and runaway adolescents tend to have sexual intercourse while using drugs and alcohol [6].

Pregnancy is a common problem faced by runaway female adolescents, and their lifetime rate for homeless girls amounts to 35% to 45% [9]; unfortunately, 40% to 70% of homeless adolescents are known to have unprotected sexual intercourse [7]. According to 2016 national data among Korean adolescents, the contraception usage rate of adolescents who have experienced sexual intercourse was found to be only 51.9% [10]. The experience rate of sexual intercourse by female at-risk adolescents (62.3%) was higher than their male counterparts, and fewer female participants (21.6%) than male participants (39.2%) answered that no contraception was used [11].

According to Whang et al. [1], at-risk adolescents who had experienced unwanted pregnancy or childbirth problems showed a higher ratio of 3.6% as compared with a ratio of 0.3% among non-at-risk adolescents. In addition, the proportion of adolescents who participated in the sex trade was found to be as high as 4.6% as compared with the rate of 0.4% found among non-at-risk adolescent; furthermore, the rate of sexual violence was found to be high at 3.3% as compared with a rate of 0.6% observed among non-at-risk adolescents. In fact, a survey of 103 Korean female teenagers (aged 13 to 18 years) who had been introduced into the sex trade [11] found that 84.4% of adolescents aged under 19 years had life experiences as runaways; this finding indicated that there was a close association between adolescents’ runaway status and sexual problems.

Meanwhile, according to the 2016 comprehensive survey of Korean adolescents’ contact with media usage and harmful environments [11], 41.5% of teenagers answered that they had seen adult videos and that their main channels for accessing adult videos was the most common with 27.6% of Internet portal sites. The 2016 national survey of adolescent risk behaviors [1] reported that 6.0% of female adolescents were found to be Internet and Smartphone addicts, which was about twice as high as the 3.2% found among male students; this finding showed the potential risks posed by exposure to risky behaviors among female adolescents. Therefore, considering that exposure to harmful environments, such as adult videos and harmful Internet sites, is a mediator for adolescents’ misbehaviors, and careful attention should be payed to the sexual health of runaway female at-risk adolescents [12]. Furthermore, social and environmental factors, such as access to harmful environments, can create social problems, which, in turn, can induce runaway behaviors [2,12].

South Korean researchers have recently reported the formation of the so-called “fam” culture centering on runaway adolescents, who contact each other through social networking services and move about in groups; these conditions could lead to organized crime-related behavior because of the predominance of intelligent, group-conscious, and violent adolescents who participate in juvenile crime [13]. Therefore, the South Korean government established adolescent safety net teams to identify and support at-risk adolescents; furthermore, it announced plans to strengthen community-based support for at-risk adolescents by establishing an integrated management system and expanding customized services for at-risk adolescents. In particular, exposure to harmful environments among runaway at-risk female adolescents increases the risk of sex-related problems, such as unwanted pregnancy and exposure to sexually transmitted diseases due to indiscriminate sex and sex trafficking, and also increases social problems such as unwed motherhood. Furthermore, since it could negatively affect adult reproductive health in the future and lead to problems such as pelvic inflammatory disease, miscarriage, infertility, or ectopic pregnancy [14], it is necessary to explore the various factors related to adolescents’ sexual experiences and establish an active management system. Therefore, it is necessary to identify the psychological characteristics of at-risk adolescents, the environment surrounding adolescents, that is, the impact of hazards that occur under changing circumstances, especially, the sexual experience. It is important to mediate environmental factors as factors related to the sexual experience of at-risk adolescents and to establish a preventive and educational system that can foster the ability to protect themselves from harmful environmental effects.

### Purpose of Research

This study aimed to identify factors related to the sexual intercourse experiences of runaway female at-risk adolescents, and also to provide basic data for preparing programs to help promote their sexual health by analyzing factors related to their sexual intercourse experiences. Toward this end, this study utilized statistical source data from the “2016 survey of Korean adolescents’ contact with media usage and harmful environments.” We used general characteristics (age, smoking, drinking experience, and hallucinogenic substance use experience), runaway experience (runaway frequency and duration), and exposure to harmful media and environments as independent variables and sexual intercourse experience as an outcome variable.

## 2. Materials and Methods

### 2.1. Research Design

This study was a secondary data analysis study that utilized statistical data from the “2016 survey of Korean adolescents’ contact with media usage and harmful environments [11]” in order to identify factors related to the sexual intercourse-related experiences of runaway female at-risk adolescents aged from 10 to 18.

### 2.2. Subjects

This study utilized data from the “2016 survey of Korean adolescents’ contact with media usage and harmful environments” (releasing and management regulations for raw data), which was conducted by the Ministry of Gender Equality and Family (MOGEF) and the National Adolescent Policy Institute (NYPI); this research aimed to secure basic data that could be used for establishing adolescent protection policies in order to protect adolescents from potentially harmful environments by identifying adolescents’ contact status with regard to harmful environments in Korea. As of 2016, there was no official population data regarding at-risk adolescents available, and probability sampling, in this regard, was impossible because the number of people institutionalized tends to fluctuate depending on the characteristics of adolescent protection facilities. Therefore, a target sample consisting of 2000 adolescents from juvenile detention centers, probation centers, and shelters was selected as the sample population, and non-probability sampling (purposive sampling) was conducted on the sample population. Out of 1876 at-risk adolescents selected for this study, 430 were female students; among these, data from 182 subjects who had experienced life as runaways (excluding those with missing values) were utilized in the analysis. Since the collected statistical data were tagged with a confidential and unidentifiable unique number, the personal information of the subjects was not gathered, and therefore the study maintained their anonymity and their information’s confidentiality. Furthermore, this research was conducted after IRB exemption was approved by Chosun University, where the researcher works (IRB no. 2-1041055-AB-N-01-2018-55).

### 2.3. Variables

This study utilized variables that were measured in the “2016 Comprehensive survey of Korean adolescents’ contact with media usage and harmful environments” as a secondary data analysis source.

#### 2.3.1. General Characteristics

The variable of age was measured and the responses for “smoking experience,” “drinking experience,” and “hallucinogenic substance use experience” were divided into two response categories, i.e., yes and no.

#### 2.3.2. Runaway Experience

The responses regarding runaway frequency in the past year were divided into the following categories: once, twice, three to four times, and five times or more. The duration of the most recent runaway event was measured by dividing the duration as follows: one day, two to five days, six to nine days, ten to thirty days, and more than one month.

#### 2.3.3. Exposure to Harmful Media and Environments

The extent of exposure to harmful media was measured using two items regarding the use of “adult images and adult publications” (which was marked “not available for adolescents” and “audience over 19”) in the past year and one question regarding the use or lack of use of “conditional meeting messengers or chat apps,” where responses were categorized as follows for each variable: yes and no. The scores for three items were calculated using the following categories: yes and none. This measure was applied to each question, and a higher score indicated greater exposure to harmful media.

The extent of the participants’ exposures to harmful environments was measured using questions about their usage of “video rooms/DVD rooms, entertainment/karaoke bars, night clubs/music clubs, and multi-rooms/room cafés”; responses to these items were measured using the categories, yes and no. The scores for these four items were calculated using the following categories for each question: yes and none. A higher score was associated with a greater likelihood of exposure to harmful environments.

#### 2.3.4. Sexual Intercourse Experience

Responses for items regarding sexual experience were measured using the following categories: yes and no. The responses for the items regarding “the first sexual intercourse” among questions related to sexual intercourse experience status were categorized into “elementary school” (age 10–12), “middle school” (age 13–15), and “high school” (age 16–18).

The responses for items regarding usage of contraceptives during sexual intercourse were categorized into ”always”, ”mostly”, ”sometimes”, and ”none”. The responses to items regarding “experience of sex education” were measured by ”yes” and ”no’. With regard to sexual victimization, the responses for items regarding “experience of attempted forced sexual intercourse” were measured by ”yes” and ”no”.

### 2.4. Analysis

The collected data were analyzed using the SPSS 26.0 WINDOW PROGRAM, and the statistical significance level was established as *p* < 0.05. Differences, in terms of sexual intercourse experience, based on general characteristics and research variables (runaway experience (frequency, duration) and exposure to harmful media and environments) were calculated using the relevant real numbers, percentages, means, and standard deviations. The following tests were also conducted: *x*² -test and Fisher’s exact test for categorical variables and *t*-test for continuous variables. Factors that could influence the sexual intercourse experiences of runaway female at-risk adolescents were analyzed using hierarchical logistic regression.

## 3. Results

### 3.1. The General Characteristics of Runaway Female At-Risk Adolescents

With regard to age, 80.2% of the subjects (146 subjects) were aged from 16 to 18 years, and 19.8% (36 subjects) were aged from 13 to 15 years. A total of 94.5% (172) of runaway female at-risk adolescents had drinking experience, and 90.1% (164) had smoking experience, while 90.1% (164) had no experience of hallucinogenic substance use.

Among the surveyed runaway female at-risk adolescents, 130 (72.5%) had sexual intercourse experience, and the demographic distribution of first sexual experiences among the survey participants were as follows: 69.2% (90) had their first experience in middle school, 22.3% (29) in high school, and 8.5% (11) in elementary school. The responses regarding contraception usage during sexual intercourse were as follows: 30.8% (40 people) answered “sometimes,” 26.9% (35) answered “not at all,” 23.1% (30) answered “mostly,” and 19.2% (25) answered “always.”

Among the runaway female at-risk adolescents, 77.5% (141) had received sex education and 6.0% (11) had experienced forced sexual intercourse attempts.

General characteristics with regard to sexual intercourse experience showed statistically significant differences in terms of age (*χ*^2^ = 7.649, *p* = 0.006), drinking experience (*χ*^2^ = 8.899, *p* = 0.006), smoking experience (*χ*^2^ = 10.364, *p* = 0.004), and hallucinogenic substance use (*χ*^2^ = 7.990, *p* = 0.002). Among subjects in the 16–18 age group, 76.0% had sexual intercourse experience. Thus, this age group had a statistically higher significance as compared with the 13–15 age group, in which 52.8% of subjects had sexual intercourse experience. In addition, the sexual experience rate among female adolescents who had experience in drinking, smoking, and hallucinogenic substance use was significantly higher than that among non-experienced subjects (Table 1).

### 3.2. Differences, in Terms of Sexual Intercourse Experience, among Runaway Female At-Risk Adolescents Based on Runaway Frequency and Duration

Analysis of sexual intercourse experiences based on runaway frequency and duration showed that there was a statistically significant difference in terms of runaway duration (*χ*^2^ = 17.40, *p* = 0.037). Among female at-risk adolescents who were found to have sexual experiences, 79.8% had a runaway duration of 30 days or more, 66.7% had a runway duration of 10 to 29 days; 50.0% had a runway duration of one day; 47.1% had a runaway duration of two to five days; and 46.2% had a runway duration of six to nine days (Table 2).

### 3.3. Differences, in Terms of Level of Exposure to Harmful Media and Environments, among Runaway Female At-Risk Adolescents Based on Sexual Intercourse Experience

The subjects’ level of exposure to harmful media was calculated as 1.21 ± 1.08 points (range of 0 to 3 points), while their level of exposure to harmful environments was calculated as 1.23 ± 1.27 points (range of 0 to 4 points). Analysis of the differences in variables (based on sexual intercourse experience) showed that exposure to harmful media (*t* = 3.61, *p* <0.001) and exposure to harmful environments (*t* = 5.79, *p* < 0.001) were statistically and significantly higher among runaway female at-risk adolescents who had sexual intercourse experiences as compared with those without such experiences (Table 3).

### 3.4. Factors Associated with Sexual Intercourse Experiences among Runaway Female At-Risk Adolescents

The control between variables is very important for including various variables, and logistic regression analysis can be used to compare the degree of effect on outcome variables when different categories are calibrated or not calibrated. Among them, hierarchical logistic analysis can be used to compare the degree to which the result variables are explained when different levels of variables are added hierarchically. The variation inflation factor (VIF) between independent variables showed the maximum value of 1.25 with less than 10, while tolerance showed the lowest value of 0.68, with a significant excess of 0.2; no problem was observed in the multicollinearity.

Model 1, which facilitated hierarchical logistic regression analysis using the demographic characteristics that showed significant differences, in terms of univariate analysis, in order to identify factors related to the sexual intercourse experiences of runaway female at-risk adolescents, showed statistical significance with regard to age and smoking. In the logistic regression model, Cox and Snell R^2^ showed how many independent variables explained the dependent variables. However, since Cox and Snell R^2^ had a maximum value of less than 1, the Nagelkerke R^2^ value that is corrected to a maximum value of 1, could be used. The Cox and Snell R^2^ value was 0.199, while the Nagelkerke R^2^ was 0.285 (Table 3).

In Model 2, which included runaway experience and exposure to harmful media and harmful environments, all existing independent variables were significant, and exposure to harmful environments had a statistically significant effect. The Cox and Snell R^2^ value was 0.296, while the Nagelkerke R^2^ was 0.426. All regression models passed the model fit tests (Wald F 31.19~31.77, *p* < 0.001) (Table 3).

Runaway female at-risk adolescents in the 16–18 age group showed a 3.52 times (95% Cl 1.34–9.26) higher amount of sexual intercourse experiences than those in the 13–15 age group; Sexual intercourse experience was 4.56 times higher (95% Cl 1.28–16.18) when smoking, and 1.97 times higher (95% Cl 1.30–2.98) when exposed to harmful environments (Table 4).

All models were additionally adjusted to account for the baseline factors of hallucinogenic substance use and forced intercourse experience.

## 4. Discussion

This study identified that the main factor related to runaway female adolescents’ sexual intercourse experiences was age. Adolescence can be largely divided into the following stages: early (10 to 13 years), mid (14 to 16 years), and late (17 to 19 years) [15]. This study found that late-stage adolescents (aged from 16 to 18) had a 3.52 times higher amount of sexual intercourse experiences than the mid-stage adolescents (aged from 13 to 15). This finding is similar to those reported by Boyer [16], where 20% of 15-year-olds and 65% of 18-year-olds were found to have had sexual intercourse. Our study’s findings also agree with the 2016 Ethiopian Demographic and Health Survey (EDHS), which reported that 24% of women started having sex before the age of 15, and 62% did so before the age of 18 [17].

A 2016 study [18] reported a steady increase in the amount of sexual experiences among adolescents in the 14–18 age group and argued that adolescents who began to have sexual intercourse at an early age were more likely to engage in dangerous sexual behaviors, such as having multiple sexual partners or practicing inaccurate or inconsistent use of condoms; these are behaviors that increase the risk of exposure to many sexual and reproductive health problems. Consequently, such behaviors can increase the risks of sexually transmitted infections (STIs) such as HIV/AIDS, unwanted pregnancies, unsafe abortions, early childbirths, and related psychosocial problems. One 2013 study showed that two-thirds of STIs that were diagnosed among patients in the 14–23 age group each year were found to be HPV infections; these infections can cause cervical or other cancers in the future [19]. Since this problem is the greatest threat to the health and well-being of adolescents [20], special caution is necessary. In particular, because adolescents who have experienced life as runaways can suffer from problems related to their peer, school, or family lives, it is necessary to assess and manage their problems while considering various aspects.

Smoking was identified as a significant variable influencing the sexual intercourse experience of runaway female at-risk adolescents; the amounts of such experiences were 4.56 times higher among adolescents who smoked than those who did not practice smoking behaviors. This finding agrees with those of a previous study, where smoking among adolescents was associated with a 2.01 times higher amount of sexual experiences, and another study that reported that smoking was associated with early sexual initiation [21,22]. The present study’s finding is also consistent with a 2008 report [23], which suggested that adolescents’ runaway status increased their risk of sexual victimization and could be associated with smoking.

According to a 2016 study [1], 29.8% of at-risk adolescents were exposed to smoking as compared with 1.7% of non-at-risk adolescents, thus, showing about 15 times higher risk of smoking. In addition, another study reported that the risk of smoking was high among both male and female adolescents who had runaway experience [24]. According to one survey that examined the differences, in terms of smoking behaviors, among adolescents who received foster care, both male and female adolescents started smoking at similar ages and showed similar rates of lifetime and current smoking [24]. However, it also reported that smoking experiences were more common among victims of sexual abuse and female victims of non-sexual abuse; this tendency resulted in significant differences, in terms of sex-related factors, between risk factors associated with smoking among male and female adolescents. In other words, since runaway female adolescents are more likely to face sexual health threats than male runaway adolescents, careful consideration of the sexual health problems they can face is necessary, and appropriate preventive interventions should be made available to them. In particular, since female adolescents can live under the care of institutions (such as probation centers or shelters) where they can receive insufficient tolerance for smoking or support for quitting smoking, active management is necessary for aiding them. Incorporating the cultural, psychological, and environmental factors associated with smoking behavior from a social-ecology perspective, interdependencies between various risks should be considered [25]. Therefore, schools and community health professionals should examine the emotional and psychological status of runaway female at-risk adolescents and provide them with education to help them develop their own internal strengths; it is important to help them gain proper awareness, which, in turn, can help them cope with sexual health challenges they could face. Furthermore, inclusive intervention that can prevent or reduce their exposure to repetitive risky behaviors is necessary to improve their sociocultural awareness. It is necessary to prepare an active management system for integrating cooperation and support among all sectors including individual, family, school, and community, along with social efforts to establish the right sex culture among adolescents.

In this study, exposure to harmful environments was a factor that affected the experience of runaway female at-risk adolescents; female at-risk adolescents who were exposed to harmful environments had a 1.97 times higher amount of sexual intercourse experiences as compared with those who had not experienced such exposures. This finding agrees with those of previous reports that reported that harmful environmental variables were positively correlated with juvenile delinquency [26] and that harmful environments could induce deviant behaviors among adolescents [12]. The use of pubs among Korean at-risk adolescents was reported to be 48.4%, which is 12.8 times higher than the 3.8% figure observed among non-at-risk adolescents; the use of entertainment and karaoke bars was 21.5%, 18 times higher than that observed among non-at-risk adolescents (1.2%); and the use of multi-rooms/room cafes was 20.5%, indicating that exposure to harmful environments had reached a serious level (one out of every five at-risk adolescents had experienced exposure to risky environments) [11].

Korea’s transition to an information society has also influenced adolescents’ formation of the “runaway fam” after the emergence of the runaway phenomenon, thus providing such adolescents with opportunities to connect with a wider range of peers beyond time and space restrictions [27]; this factor also helped to increase such adolescents’ exposure to various harmful environments [6]. Therefore, considering that at-risk adolescents have a relatively higher exposure to harmful environments than non-at-risk adolescents [28], it is necessary to analyze the characteristics of users and the characteristics of the harmful environments that influence adolescents’ risky behaviors in order to raise awareness about the negative effects of continuous exposure to harmful environments. It is also suggested that practical education, including preventing exposure to harmful environments and the promotion of sex education and sex counseling, should be conducted by establishing various information linkages and cooperation within the school curriculum after considering accessibility and efficiency.

Statistical data used in this study is difficult to be generalized because of the following limitations. This study was a secondary analysis of the data from a Korean national sample survey, and thus only focused on the original variables such as smoking, drinking, and drugs, without considering additional variables in a wider range such as addictive substances. This study also failed to measure related variables in utilization of development tools whose reliability and validity have been verified.

In order to promote sexual health of runaway female at-risk adolescents, active education and intervention is necessary. This can help to reduce their exposure to harmful elements that include smoking and harmful environments. Furthermore, as a continual educational intervention, it is important to promote sexual education programs to help runaway female at-risk adolescents pursue safer sexual behaviors and recognize personal factors threatening their sexual health such as smoking, drinking, and unprotected sexual intercourse. In addition, it should be perceived that the exposure to harmful environments and media can negatively affect the sexual health. Above all, social well-being programs aimed at runaway female at-risk adolescents should prioritize ways to help them to develop their capacity to protect themselves from the effects of harmful environments. Toward this end, school health teachers and community healthcare providers should be aware of the environments frequented by at-risk adolescents; thus, they require training to help them deal with such adolescents’ problems actively and proactively.

## 5. Conclusions

This study aimed to identify factors that are related to sexual intercourse experiences among runaway female at-risk adolescents in order to create interventions aimed at improving their daily lives in terms of sexual health promotion. Consequently, age, smoking, and exposure to harmful elements, including media and environments, were identified as factors that could influence such adolescents’ sexual intercourse experiences; thus, it was confirmed that the complex issue of sexual intercourse experience was associated with social and environmental factors such as smoking and exposure to harmful environments, as well as personal characteristics such as age.

This study found that smoking and sex-related problems among runaway female at-risk adolescents could act as risk factors for adult sexual health in the future; therefore, schools should establish direct and indirect channels for assessing the sex-related problems of runaway female at-risk adolescents. Furthermore, it is important to establish proactive and preventive measures for promoting their sexual health. In order to minimize adolescents’ exposure to harmful environments, a social cooperation system, including schools, family, local community, community health centers, and social workers, should be established for assessing, and mediating within, the school environment. In particular, it is necessary to provide continuous support at the family level so that at-risk adolescents can recognize and make efforts to proactively change their lives. Lastly, this study suggests that further studies should be conducted to deal with various variables related to individuals, families, schools, and communities in the future; it also recommends the development of programs to help promote sexual health by identifying factors related to risk behaviors among female at-risk adolescents.

## Figures and Tables

**Table 1:** General characteristics based on sexual intercourse experience (*N* = 182).

Variables	Categories	Total Sample (*n* = 182)		Sexual Intercourse Experience				*x* ^2^	*p*
				Yes (*n* = 130)		No (*n* = 52)			
		*n* (%)	M ± SD	*n* (%)	M ± SD	*n* (%)	M ± SD		
Age (yr)	13–15	36 (19.8)	15.69 ± 0.52	19 (52.8)	15.68 ± 058	17 (47.2)	15.71 ± 0.47	7.649	0.006
	16–18	146 (80.2)	18.06 ± 0.96	111 (76.0)	18.05 ± 0.95	35 (24.0)	18.11 ± 1.02		
Drinking	Yes	172 (94.5)		127 (73.8)		45 (26.2)		8.899	0.006
	No	10 (5.5)		3 (30.0)		7 (70.0)			
Smoking	Yes	164 (90.1)		123 (75.0)		41 (25.0)		10.364	0.004
	No	18 (9.9)		7 (38.9)		11 (61.1)			
Hallucinogenic substance use	Yes	18 (9.9)		18 (100.0)		0 (0.0)		7.990	0.002
	No	164 (90.1)		112 (68.3)		52 (31.7)			
First sexual intercourse	Elementary school (age 10–12)			11 (8.5)					
	Middle school (age 13–15)			90 (69.2)					
	High school (age 16–18)			29 (22.3)					
Contraception use	Always			25 (19.2)					
	Mostly			30 (23.1)					
	Sometimes			40 (30.8)					
	Not at all			35 (26.9)					
Sex education	Yes	141 (77.5)		105 (74.5)		36 (25.5)		2.833	0.070
	No	41 (22.5)		25 (61.0)		16 (39.0)			
Experience of forced intercourse	Yes	11 (6.0)		11 (0)		0 (0.0)			0.035
	No	171 (94.0)		119 (69.6)		52 (30.4)			

**Table 2:** Sexual intercourse experience based on runaway frequency and duration (*N* = 182).

Runaway Severity		Total Sample (*n* =182)	Sexual Intercourse Experience		*x* ^2^	*p*
			Yes (*n* = 130)	No (*n* = 52)		
		*n* (%)	*n* (%)	*n* (%)		
Frequency	1	46 (25.3)	30 (65.2)	16 (34.8)	3.81	0.282
	2	36 (19.8)	23 (63.9)	13 (36.1)		
	3–4	29 (15.9)	21 (72.4)	8 (27.6)		
	≥5	71 (39.0)	56 (78.9)	15 (21.1)		
Duration (day)	1	6	3 (50.0)	3 (50.0)	17.40	0.037
	2–5	17	8 (47.1)	9 (52.9)		
	6–9	13	6 (46.2)	7 (53.8)		
	10–29	21	14 (66.7)	7 (33.3)		
	30	124	99 (79.8)	25 (20.2)		

**Table 3:** Research variables based on sexual intercourse experience (*N* = 182).

Variables	Range	Total Sample (*n* = 182)	Sexual Intercourse Experience		*t*	*p*
			Yes (*n* = 130)	No (*n* = 52)		
		M ± SD *	M ± SD	M ± SD		
Harmful media	0~3	1.21 ± 1.08	1.39 ± 1.08	0.76 ± 0.93	3.61	<0.001
Harmful environments	0~4	1.23 ± 1.27	1.55 ± 1.28	0.44 ± 0.80	5.79	<0.001

* M ± SD = Mean ± standard deviation.

**Table 4:** Logistic regression predicting runaway female at-risk adolescents’ sexual intercourse experience levels (*N* = 182).

Categories				Model 1 *				Model 2 ^†^			
				OR	95% CI		*p*	OR	95% CI		*p*
					Low	High			Low	High	
**Constant**		Reference	Item	-	-	-	0.004	-	-	-	0.005
Age (yr)		14–16	17–19	3.94	1.66	9.34	0.002	3.52	1.34	9.26	0.011
Drinking		No	Yes	2.36	0.51	10.97	0.275	1.65	0.32	8.61	0.552
Smoking		No	Yes	4.82	1.53	15.16	0.007	4.56	1.28	16.18	0.019
Runaway period (day)		1						1.85	0.28	12.32	0.524
Exposure to harmful elements	Harmful media							1.41	0.94	3.11	0.097
	Harmful environments							1.97	1.30	2.98	0.001

* Nagelkerke R^2^ was 0.285 (Cox and Snell R^2^ was 0.199); ^†^ Nagelkerke R^2^ was 0.426 (the Cox and Snell R^2^ value was 0.296). OR, odds ratio and CI, confidence interval.
